# Complete Genome Sequences of Two Geographically Distinct *Legionella micdadei* Clinical Isolates

**DOI:** 10.1128/genomeA.00436-17

**Published:** 2017-06-01

**Authors:** Amy J. Osborne, Bethany R. Jose, Jasper Perry, Zoe Smeele, Jack Aitken, Paul P. Gardner, Sandy Slow

**Affiliations:** a School of Biological Sciences, University of Canterbury, Christchurch, New Zealand; b Department of Pathology, University of Otago Christchurch, Christchurch, New Zealand

## Abstract

*Legionella* is a highly diverse genus of intracellular bacterial pathogens that cause Legionnaire’s disease (LD), an often severe form of pneumonia. Two *L. micdadei* sp. clinical isolates, obtained from patients hospitalized with LD from geographically distinct areas, were sequenced using PacBio SMRT cell technology, identifying incomplete phage regions, which may impact virulence.

## GENOME ANNOUNCEMENT

*Legionella* is a highly diverse genus of intracellular bacterial pathogens that can infect human lung macrophages, causing an often severe form of pneumonia known as Legionnaire’s disease (LD). While *L. pneumophila* and *L. longbeachae* spp. are responsible for the majority of LD, improved diagnostic testing (PCR-based assays, which detect all species) has found cases caused by other species, such as *L. micdadei*, which is thought to be responsible for about 60% of LD cases not related to *L. pneumophila* or *L. longbeachae* (1). These have been predominantly isolated from immunocompromised patients (2, 3). Recently, a complete prophage sequence was identified in the *L. micdadei* ATCC 33218^T^ genome, yet the prophage is absent from a separate Australian *L. micdadei* clinical isolate (4). Despite this, sequencing of *L. micdadei* strains has so far shown high genomic synteny, suggesting that *L. micdadei* genomes are highly conserved, except for their mobilome, which may vary for geographically distinct strains (4). Considering this, investigating the diversity within *L. micdadei* may contribute toward identifying mobilome features that may have strong implications for revealing the origin of a strain. Thus, to further elucidate the genomic diversity of *Legionella* spp. associated with LD, the full genomes of two geographically distinct clinical isolates of *L. micdadei* were sequenced.

Two *L. micdadei* isolates were obtained from patient sputum: LM2015 from Christchurch (South Island, New Zealand) and LM2016 from Waikato (North Island, New Zealand). Strains were sequenced with single-molecule real-time (SMRT) technology on PacBio RSII. The Canu version 1.3 assembler was used to generate single contigs and trim reads (5, 6). Complete genomes were annotated with Prokka version 1.11 (7–10). Genomic structural rearrangements between isolates were carried out using whole-genome BLAST analysis, and results were visualized using both the Artemis Comparison Tool (ACT) (11) and the MAUVE genome comparison tool version 2.4.0 (12). Sequences were analyzed for quality by using Qualimap version 2.2.1 (13). Multiple sequence alignments between LM2015 and LM2016 were generated using Mauve (12).

With a genome size of approximately 3.3 Mb, PacBio SMRT sequencing provided approximately >98% coverage of the entire *L. micdadei* genome. Both genomes had a G+C content of 40.51%. LM2015 contained 3,038 genes, 2,977 proteins, 9 rRNAs, and 45 tRNAs. LM2016 contained 3,002 genes, 3,189 proteins, 9 rRNAs, and 44 tRNAs. Despite relatively identical genome size and content, 8,059 single nucleotide polymorphisms were identified between strains. PHASTER revealed incomplete phage DNA (a score of <70) in both LM2015 (three genomic regions) and LM2016 (two regions).

The 2015 and 2016 clinical isolates sequenced in this study were compared to the ATCC type strain genome for *L. micdadei*. Aligning these genomes revealed highly conserved genome content with some rearrangements of genomic regions. These rearrangements may be due to the insertion of mobile elements that are characteristic of the *Legionella* genus. This study expands our understanding of the diversity between strains within the species.

### Accession number(s).

These genomes have been deposited in GenBank under the accession numbers CP020614 and CP020615.
